# Supplementary material for: Values and Uncertainty at End of Life: A Standardized Patient Case for Preclinical Medical Students
Source: MedEdPORTAL. 2025 Mar 4;21:11503. doi: 10.15766/mep_2374-8265.11503 (PMC11876469; doi:10.15766/mep_2374-8265.11503)
Supplement: Supplementary file 1 — SP Case.docxPeer Debrief Questions.docxDoor Note.docxStudent Self-Assessment.docxSP Assessment.docx [file mep_2374-8265.11503-s001.zip › C. Door Note.docx]

# Door Chart/Note & Learner Instruction

**Setting (place/time)**

| **PMD office, MRI and oncologist follow-up from yesterday** |
| --- |

**Patient Name: Gordon/Sharon Phillips**

**Age: 45yo**

**Gender: M/F**

**Chief Complaint: MRI and oncologist follow-up**

**Vital Signs:**

Blood Pressure: 123/94

Temperature: 98.0F

Respiratory Rate: 16

Heart Rate: 91

BMI: 18

**Lab Results:** n/a

**Image Results:**

MRI head: redemonstration of right parietal cystic mass consistent with glioblastoma multiforme, increased in volume since last imaging after second resection

**Instructions to Learners:**

| Patient was told by their oncologist yesterday after the MRI that “there is no clear path forward” with regards to treating their cancer. In the clinic note to you the oncologist writes unambiguously that there are no established therapies or experimental trials available with a chance of curing the cancer.  The two options provided by the oncologist are   1. palliative chemotherapy in a new regimen requiring clinic visits three days a week with medication known to cause severe nausea and limb pain, with an expected prognosis of one year of life 2. comfort measures only through home hospice to focus on medication and therapy for pain control and quality of life, with an expected prognosis of two months of life   The patient will have questions about what to do. A physical exam is not necessary.   - Encounter length: 20 minute patient encounter followed by 5 minute self-assessment and 5 minute observer and peer debrief |
| --- |
