# Supplementary material for: Values and Uncertainty at End of Life: A Standardized Patient Case for Preclinical Medical Students
Source: MedEdPORTAL. 2025 Mar 4;21:11503. doi: 10.15766/mep_2374-8265.11503 (PMC11876469; doi:10.15766/mep_2374-8265.11503)
Supplement: Supplementary file 1 — SP Case.docxPeer Debrief Questions.docxDoor Note.docxStudent Self-Assessment.docxSP Assessment.docx [file mep_2374-8265.11503-s001.zip › D. Student Self-Assessment.docx]

**Appendix D – Student Self-Assessment**

to be administered to students immediately following the conclusion of the standardized patient encounter; allow five minutes to complete

|  | | **Strongly Agree (7)** | **Agree (6)** | **Somewhat Agree (5)** | **Neither Agree nor Disagree (4)** | **Somewhat Disagree (3)** | **Disagree (2)** | **Strongly Disagree (1)** |
| --- | --- | --- | --- | --- | --- | --- | --- | --- |
| *Please indicate your agreement with these general statements.* | | | | | | | | |
| 1 | I feel comfortable providing care to a patient when there are no curative treatments available. | 7 | 6 | 5 | 4 | 3 | 2 | 1 |
| 2 | I am confident I can discuss a patient’s limited prognosis with them.^a^ | 7 | 6 | 5 | 4 | 3 | 2 | 1 |
| 3 | I am confident I can account for a patient’s religious and spiritual beliefs and practices when discussing their terminal condition. | 7 | 6 | 5 | 4 | 3 | 2 | 1 |
| 4 | I am confident I can account for a patient’s cultural beliefs and practices when discussing their terminal condition.^a,b^ | 7 | 6 | 5 | 4 | 3 | 2 | 1 |
| 5 | I am confident I can admit uncertainty to a patient.^a^ | 7 | 6 | 5 | 4 | 3 | 2 | 1 |
| *Please indicate your agreement with these statements about the OSCE Exam you just completed.* | | | | | | | | |
| 6 | The time I spent with the patient was valuable to them. | 7 | 6 | 5 | 4 | 3 | 2 | 1 |
| 7 | I felt comfortable during this OSCE. | 7 | 6 | 5 | 4 | 3 | 2 | 1 |
| 8 | I felt more comfortable during this OSCE than other OSCEs I have taken. | 7 | 6 | 5 | 4 | 3 | 2 | 1 |
| 9 | I felt anxious during this OSCE. | 7 | 6 | 5 | 4 | 3 | 2 | 1 |
| 10 | I felt more anxious during this OSCE than other OSCEs I have taken. | 7 | 6 | 5 | 4 | 3 | 2 | 1 |

^a^These items reflect one or more "expected behaviors for an entrustable learner" from the AAMC's Core Entrustable Professional Activities for Entering Residency. For further information please see Association of American Medical Colleges. Core entrustable professional activities for entering residency curriculum developers’ guide. https://store.aamc.org/downloadable/download/sample/sample_id/63/. Accessed July 10, 2024.

^b^These items reflect one or more concepts from the Kalamazoo Essential Elements Communication Checklist-Adapted. No items from the checklist were used here verbatim. For further information please see Rider EA. Interpersonal and communication skills. In: Rider EA, Nawotniak RH. *A Practical Guide to Teaching and Assessing the ACGME Core Competencies*. 2nd ed. Marblehead, MA: HCPro, Inc., 2010, pp 1-137.
