# Supplementary material for: Values and Uncertainty at End of Life: A Standardized Patient Case for Preclinical Medical Students
Source: MedEdPORTAL. 2025 Mar 4;21:11503. doi: 10.15766/mep_2374-8265.11503 (PMC11876469; doi:10.15766/mep_2374-8265.11503)
Supplement: Supplementary file 1 — SP Case.docxPeer Debrief Questions.docxDoor Note.docxStudent Self-Assessment.docxSP Assessment.docx [file mep_2374-8265.11503-s001.zip › E. SP Assessment.docx]

**Appendix E – SP Assessment**

to be administered to standardized patients immediately following the conclusion of the standardized patient encounter; allow ten minutes to complete

| **STANDARD SKILLS** | | | | | | **Competently Performed (3)** | | **Needs Improvement (2)** | | **Did Not Attempt (1)** |
| --- | --- | --- | --- | --- | --- | --- | --- | --- | --- | --- |
| 1 | Establishes and maintains rapport with the patient | | | | | 3 | | 2 | | 1 |
| 2 | Maintains eye contact | | | | | 3 | | 2 | | 1 |
| 3 | Speaks clearly and audibly | | | | | 3 | | 2 | | 1 |
| 4 | Demonstrates active listening (includes non-verbals, headnodding, posture) | | | | | 3 | | 2 | | 1 |
| 5 | Demonstrates empathy when appropriate throughout the encounter | | | | | 3 | | 2 | | 1 |
| 6 | Avoids using medical jargon, or explains jargon throughout the encounter | | | | | 3 | | 2 | | 1 |
| 7 | Adjusts tone and pacing as appropriate throughout the encounter | | | | | 3 | | 2 | | 1 |
| 8 | Begins the HPI with open-ended questions | | | | | 3 | | 2 | | 1 |
| 9 | Negotiates agenda/focus of visit | | | | | 3 | | 2 | | 1 |
| 10 | Elicits patient's perspective and beliefs (fears, concerns, ideas) | | | | | 3 | | 2 | | 1 |
| 11 | Facilitates the storytelling by saying, "Tell me more" | | | | | 3 | | 2 | | 1 |
| **PALLIATIVE SKILLS** | | **Strongly Agree (7)** | **Agree (6)** | **Somewhat Agree (5)** | **Neither Agree nor Disagree (4)** | **Somewhat Disagree (3)** | **Disagree (2)** | | **Strongly Disagree (1)** | |
| 12 | The student created a mutual understanding of the patient’s prognosis.^a^ | 7 | 6 | 5 | 4 | 3 | 2 | | 1 | |
| 13 | The student delivered the bad news in very plain language, slowly and carefully.^a,b^ | 7 | 6 | 5 | 4 | 3 | 2 | | 1 | |
| 14 | The student allowed for silence as appropriate.^b^ | 7 | 6 | 5 | 4 | 3 | 2 | | 1 | |
| 15 | The student elicited the patient’s goals of care.^a^ | 7 | 6 | 5 | 4 | 3 | 2 | | 1 | |
| 16 | The student ended the encounter with a clear plan for next steps (this does not necessarily mean that the patient and student decided which of the two treatment courses to pursue).^a^ | 7 | 6 | 5 | 4 | 3 | 2 | | 1 | |
| 17 | I am satisfied with the medical care the student provided. | 7 | 6 | 5 | 4 | 3 | 2 | | 1 | |
| 18 | The student responded appropriately to the patient's emotions.^a^ | 7 | 6 | 5 | 4 | 3 | 2 | | 1 | |
| 19 | The student assured the patient that the medical team would continue to help the patient. | 7 | 6 | 5 | 4 | 3 | 2 | | 1 | |
| 20 | The student offered to partner with the patient in having difficult conversations.^a^ | 7 | 6 | 5 | 4 | 3 | 2 | | 1 | |
| 21 | The student conducted the encounter with an honest, trustworthy demeanor.^a^ | 7 | 6 | 5 | 4 | 3 | 2 | | 1 | |
| 22 | The student elicited the patient’s religious and/or spiritual beliefs and, if appropriate, incorporated these beliefs into the discussion. | 7 | 6 | 5 | 4 | 3 | 2 | | 1 | |
| 23 | The student asked about the patient’s significant relationships/support system.^a,b^ | 7 | 6 | 5 | 4 | 3 | 2 | | 1 | |
| 24 | The student considered cultural and other factors that influenced the patient’s understanding of illness/diagnosis.^a,b^ | 7 | 6 | 5 | 4 | 3 | 2 | | 1 | |
| 25 | The student demonstrated respect for the patient’s autonomy.^a^ | 7 | 6 | 5 | 4 | 3 | 2 | | 1 | |
| 26 | The student demonstrated compassion.^b^ | 7 | 6 | 5 | 4 | 3 | 2 | | 1 | |
| 27 | The student offered false hope that the condition is not imminently terminal. | 7 | 6 | 5 | 4 | 3 | 2 | | 1 | |
| 28 | The student effectively communicated both known details and uncertainty.^a^ | 7 | 6 | 5 | 4 | 3 | 2 | | 1 | |

^a^These items reflect one or more "expected behaviors for an entrustable learner" from the AAMC's Core Entrustable Professional Activities for Entering Residency. For further information please see Association of American Medical Colleges. Core entrustable professional activities for entering residency curriculum developers’ guide. https://store.aamc.org/downloadable/download/sample/sample_id/63/. Accessed July 10, 2024.

^b^These items reflect one or more concepts from the Kalamazoo Essential Elements Communication Checklist-Adapted. No items from the checklist were used here verbatim. For further information please see Rider EA. Interpersonal and communication skills. In: Rider EA, Nawotniak RH. *A Practical Guide to Teaching and Assessing the ACGME Core Competencies*. 2nd ed. Marblehead, MA: HCPro, Inc., 2010, pp 1-137.
