# Supplementary material for: Values and Uncertainty at End of Life: A Standardized Patient Case for Preclinical Medical Students
Source: MedEdPORTAL. 2025 Mar 4;21:11503. doi: 10.15766/mep_2374-8265.11503 (PMC11876469; doi:10.15766/mep_2374-8265.11503)
Supplement: Supplementary file 1 — SP Case.docxPeer Debrief Questions.docxDoor Note.docxStudent Self-Assessment.docxSP Assessment.docx [file mep_2374-8265.11503-s001.zip › A. SP Case.docx]

Appendix A: Standardized Patient Case

Date: January 2022

Primary Case Author: Ryan Jenkins

Secondary Case Author: Erin Lamb, Anastasia Rowland-Seymour

Standardized Patient Educator: Anastasia Rowland-Seymour

Name of Case: Values and Uncertainty at End-of-Life

Name of Educational and/or Assessment Activity: Values and Uncertainty at End-of-Life

Patient Name: Gordon/Sharon Phillips

Chief Complaint: did not fully understand information provided by oncologist

Most Likely Diagnosis and Differential With Rationale From History and/or Physical Exam: terminal glioblastoma multiforme; the diagnosis is provided to students in the door note and identifying it/differentiating it from others is not the purpose of the case

Challenge Question:

Patient was told by their oncologist yesterday after the MRI that “there is no clear path forward” with regards to treating their cancer. In the clinic note to you the oncologist writes unambiguously that there are no established therapies or experimental trials available with a chance of curing the cancer.

The two options provided by the oncologist are

1. palliative chemotherapy in a new regimen requiring clinic visits three days a week with medication known to cause severe nausea and limb pain, with an expected prognosis of one year of life
2. comfort measures only through home hospice to focus on medication and therapy for pain control and quality of life, with an expected prognosis of two months of life

The patient will have questions about what to do. A physical exam is not necessary.

Domains: Check all that apply

- Professionalism
- ***Communication and Interpersonal Skills***
- Medical History
- Physical Exam
- ***Shared Decision-Making***
- Patient Education
- Clinical Reasoning
- Documentation
- Handoff
- Presentation
- Other: ***Responding To Emotion***

Type and Level of Learner: second-year medical student near the end of preclinical education

Case Objectives: Please list specific objectives for each of the domains you have checked above:

1. Discuss goals and values with a patient facing a terminal diagnosis.
2. Determine a reasonable plan/next step for a patient in the setting of clinical uncertainty.
3. Communicate professionally and empathetically with a patient facing a new diagnosis of a terminal illness.

| SETTING: outpatient, in patient, ED, home, nursing home, rehab, group, etc. | PMD office, MRI and oncologist follow-up from yesterday |
| --- | --- |
| PATIENT PROFILE: Information about the “patient” that helps select an SP and helps the learner get an understanding of them as a person. SP will know more information about the patient than learner will ever ask but allows SP to portray a fully developed patient personality. If none of the items below are particulars for the case, please write “all may be used.” | |
| Age range | middle-aged (case is written as a 45-year-old patient) |
| Religious/spiritual background | The patient is Christian with church attendance a few times a month, believes in heaven but still very afraid of actually dying. This does not have to be mentioned during the encounter if there is no natural place to bring it up, however have a very low threshold to bring it up if the student asks a question that is relevant. |
| Sex (e.g., male, female, intersex, transwoman, transman) | all may be used |
| Sexual orientation (e.g., heterosexual, lesbian, gay, bisexual, pansexual, queer, asexual) | all may be used |
| Gender expression (e.g., man, woman, genderqueer) | all may be used |
| Race and ethnicity | all may be used |
| Physical description (e.g., BMI, height range) | all may be used |
| Physical limitations | The activities in which the patient finds enjoyment require lots of energy and physical activity (biking, hiking, traveling), and they are frustrated that they can no longer do them and probably won’t ever be able to again. The patient does not need to enact any specific physical limitations during the encounter itself. |
| Patient appearance (e.g., disheveled, hospital gown, business casual, casual) | casual |
| Moulage + location (e.g., none, bruises, scars, body piercing, tattoos) | none |
| Affect (e.g., pleasant, cooperative) | Affect: disappointed but interactive, one anger outburst as below  Body language: tense  Facial expression: tense  Eye contact: natural |
| Family group (e.g., who is family, who they live with) | Lives at home with spouse and 17-year-old daughter in suburban city. Parents live in Richmond VA, have flown back and forth to help as needed. |
| Education | JD |
| Level of health literacy | High |
| Employment, if any - present and past, noting any current stresses | Lawyer, although hasn’t worked since 2019 when cancer was diagnosed. |
| Home/homeless - type of dwelling, number of stories, owned or rented | Suburban home, owned. |
| Financial situation - any current stresses | Spouse supports patient and their 17-year-old daughter by working as a schoolteacher. No specific financial stressors. |
| Insurance status (e.g., un/under/insured, public/private, HMO/PPO) | Insured. |
| Habits (i.e., diet, exercise, caffeine, smoking, alcohol, drugs) | Denies drug and tobacco use.  Alcohol Use: glass of wine nightly on most nights although less so since GBM diagnosis  Diet: no specific restrictions, eats a variety of foods  Exercise: previously very active but has not had energy to exercise regularly since 2019 |
| Activities (i.e., hobbies, sports, clubs, friends) | enjoyed biking, hiking, traveling |
| Typical day - what is the usual daily routine | all may be used although note effect of symptoms on daily life as noted below |

| CASE INFORMATION | |
| --- | --- |
| Chief Concern: What the patient will say when greeted by the student. The patient’s primary reason for seeking medical care often stated in their own words. | Well I got the results of my latest head scan yesterday and I don’t know what to do about them. The oncologist said he doesn’t know what to do either. Do you have any ideas? |
| Additional Concerns: Other, if any, concerns the patient has today (i.e., symptoms, requests, expectations, etc.) that will become part of set agenda. | Daughter graduates high school in five months. You would like to attend this graduation if possible, though you’re not sure at what cost and want to weigh this against the risk of pain and debilitation from life-prolonging therapy. |
| THE PATIENT’S STORY: The SP will be asked to tell their symptom story and the personal and emotion impact for each of their concerns. You will want to write this in the patient’s voice. The symptom story should be able to answer this question: “Tell me more about [chief concern/additional concern], starting at the beginning and bringing me up to now.”  The personal context should be able to answer questions concerning the broader personal/psychosocial context of symptoms, especially the patient’s beliefs/attributions.  The emotional context should be able to ask how are you doing with this, how does this make you feel, how has this affected you emotionally? IMPACT: How has this affected your life? How has this been for your family? | 45y/o no significant PMH who was diagnosed with right parietal glioblastoma multiforme in 2019, status post resection and radiation therapy in 2019. No residual focal neurologic deficits although has been fatigued and weak since then. Had to quit job as a lawyer at that time and been unable to return. In November 2021 began having daily worsening headaches and blurry vision, repeat MRI showed recurrence and spread of mass, underwent repeat resection and radiation. New scans show persistence of tumor in unresectable location despite radiation therapy. Patient had an oncology appointment yesterday and was told “there’s no clear path ahead,” but wants to discuss what this means with their PMD.  The patient has been through multiple rounds of chemotherapy of various types with side-effects primarily of nausea and hair loss. After the cancer first went into remission they were told it had an approximately 50% chance of recurring. Before their most recent surgery they were told there was an approximately 50% chance of the surgery allowing the tumor to then be cured with more chemo. The patient understands that their condition is serious and had the strong potential of progressing to terminal, but does not realize until talking to the student that now is the point at which there is no longer the possibility of a curative outcome.  Information offered spontaneously (what the patient can disclose after an open-ended question)   - I love my family and want to be there for them as much as possible. - I want the time to be good time. I’m scared of being in more pain, especially pain that’s worse than what I have now. - Can discuss the symptoms, the emphasis should be that they negatively affect your quality of life. If the student asks specific questions trying to diagnose the underlying physiology, aim for vagueness. - The activities in which the patient finds enjoyment require lots of energy and physical activity, and they are frustrated that they can no longer do them and probably won’t ever be able to again. - The patient has not shared with their daughter that their cancer may be a fatal diagnosis. In fact, the family never discusses what it means to die and has not taken steps to be prepared for that possibility.   Door note provides an MRI result as follows:  MRI head: redemonstration of right parietal cystic mass consistent with glioblastoma multiforme, increased in volume since last imaging after second resection |
| HISTORY OF PRESENT ILLNESS: Although some of the HPI will be given in the patient’s symptom story, the learners will expand the story during the direct question section. Below, describe the detailed history, usually about the chief concern, which the student must develop in order to make a useful assessment of the problem: | |
| Onset (when; gradual or sudden) | several weeks ago although even before the scan was fatigued/mildly uncomfortable at baseline |
| Setting (what was going on or where was patient when symptoms first noticed?) | nonspecific |
| Duration (how long) | never entirely gone but spikes during the day at random intervals |
| Time relationships (frequency, constant or intermittent) | daily, of varying intensity |
| Location | bilateral frontotemporal |
| Radiation | entire head |
| Quality | diffuse headache and nausea |
| Amount | pain score baseline 3-4 with spikes to 9-10 |
| Aggravated by what | unclear, no particular pattern |
| Relieved by what | lying still, sometimes additional PRN oxycodone |
| Associated with what | blurry vision when reading/watching TV |
| Attitude (what does the patient think is the problem, and how do they feel about it) | The pain is always there, has been there for several months, and patient has learned to cope with the current quantity but is very anxious that it could get worse. Relies on daily oxycodone and about half of days has to take an additional PRN dose. Patient understands that it’s from the brain tumor and doesn’t expect the PMD to improve it in this visit but wants to make sure this is addressed by whatever overall plan of care they develop. The nausea is also always there but less worrisome to the patient; their appetite is not great anyway. |
| Overall course | worsening |
| REVIEW OF SYSTEMS: Significant positives and negatives | |
| n/a |  |
|  |  |
|  |  |
|  |  |
|  |  |
| Past medical history |  |
| Medication allergies (name and reaction) | none |
| Environmental allergies (name and reaction) | none |
| Illnesses | right parietal glioblastoma multiforme diagnosed in 2019, s/p resection and radiation in 2019 and 2021 |
| Vaccinations | up to date |
| Surgeries | GBM resection x2 |
| Accidents/injuries/trauma | none |
| Hospitalization | multiple for surgery and radiation 2/2 GBM |
|  | |
| Inclusive sexual and reproductive history | |
| Sexual practices  Sexual partners  Protection: Use of safer sex practices  Use of birth control if appropriate  Risk of intimate partner violence | sexually active exclusively with spouse without using protection, no safety concerns |
| OB/GYN history | (if female) G1P1001, uncomplicated term SVD at 28yo  Age of onset of menses: any may be used  Age of menopause: not happened yet  Number of pregnancies: 1  Number of live births: 1  Number of miscarriages: 0  Number of abortions: 0 |
| Medications | Prescription/dose/reason: see below  Over the counter/dose/reason: n/a  Herbs/supplements/dose/reason: n/a  Other:  oxycodone sched and PRN, ondansetron PRN (“I don’t remember what dose, my wife/husband gives it to me”) |
| Immunizations | up to date |
| Tobacco products:   - Cigarettes - Cigar - Pipe - Chew - E-cigarettes | - ***Never*** - Past - year started/year quit - Current   - Quantity   - # of years |
| Alcohol   - Beer - ***Wine*** - Liquor - Other | - Never - Past - year started/year quit - ***Current***   - Quantity: 1 glass per night on most nights although less so since GBM diagnosis   - # of years: many |
| Drugs   - Weed - Cocaine - Heroin - Meth - IV - Inhalants - Other | - ***Never*** - Past - year started/year quit - Current   - Quantity   - # of years |
| Diet (describe) | no specific restrictions, eats a variety of foods |
| Exercise (describe) | previously very active but has not had energy to exercise regularly since 2019 |
| List any other important social history or information important to this case | n/a |
| Family history |  |
| Mother, father, siblings, grandparents, and other significant findings | n/a |
|  |  |
| Physical Exam - List exam maneuvers expected for this case and any abnormal findings that SP will simulate. (tenderness, hyper-hypo reflex, rebound, weakness, etc.)  physical exam not expected to be performed | |
| PHYSICAL EXAM FINDINGS |  |
| 1. Written in layperson’s terms | n/a, physical exam not expected to be performed |
| 1. General appearance - affect, appearance, position of patient at opening (i.e., sitting, lying down, holding abdomen, etc.) | sitting, neutral at opening |
| 1. Vital signs | Blood Pressure: 123/94  Temperature: 98.0F  Respiratory Rate: 16  Heart Rate: 91  BMI: 18 |
| 1. Specific findings and affect | n/a, physical exam not expected to be performed |
| 1. Response to certain physical movements | n/a, physical exam not expected to be performed |
|  |  |
| DIAGNOSIS AND DIFFERENTIAL |  |
| Diagnosis with support from positive and negative history and PE findings | n/a, diagnosis is known at start of case |
| Differential with support from positive and negative history and PE findings | n/a, diagnosis is known at start of case |
|  |  |
| MANAGEMENT OR DIAGNOSTIC PLAN | Patient should say “How do I even make a decision like this?” with respect to picking a treatment course.  The patient should expect a discussion of goals of care, patient’s values and preferences, and some concrete next steps to take. Note that this does not mean the patient has to have chosen which of the two paths to take, just that they have a plan as to what to do next. It’s OK for the plan to be wanting to talk more with your family at home before making a decision about which option. If the student gets stuck before coming up with a plan you can prompt with “So what’s the next step?” |
|  |  |
| PROFESSIONALISM ISSUES OR CHALLENGES | At one point while discussing goals the patient should become abruptly angry and shout something to the effect of: “Goddammit, why can’t they just cut it out? I want to be around for all of this stuff!” Anger should not be directed at the medical student but instead at the situation. Note the student’s response and allow your anger to be defused relatively easily if you think they react appropriately (i.e. remaining calm, responding with empathy). |
