# Supplementary material for: Values and Uncertainty at End of Life: A Standardized Patient Case for Preclinical Medical Students
Source: MedEdPORTAL. 2025 Mar 4;21:11503. doi: 10.15766/mep_2374-8265.11503 (PMC11876469; doi:10.15766/mep_2374-8265.11503)
Supplement: Supplementary file 1 — SP Case.docxPeer Debrief Questions.docxDoor Note.docxStudent Self-Assessment.docxSP Assessment.docx [file mep_2374-8265.11503-s001.zip › B. Peer Debrief Questions.docx]

**Appendix B – Peer Debrief Questions**

to be filled out by peer observers during the encounter and then referred to as needed during peer debrief with students at the end of the workshop (after students complete their self-assessment in Appendix D)

| **TERMINAL PATIENT ENCOUNTER** | | | | |
| --- | --- | --- | --- | --- |
| **OPENING VISIT** | | **Competently Performed (3)** | **Needs Improvement (2)** | **Did Not Attempt (1)** |
| **1** | Knocks and enters the room | 3 | 2 | 1 |
| **2** | Sanitizes hands upon entering the room | 3 | 2 | 1 |
| **3** | Shakes hands and **introduces self** by first and last name | 3 | 2 | 1 |
| **4** | States that they are a medical student | 3 | 2 | 1 |
| **5** | Asks patient how they would like to be addressed, including formal title | 3 | 2 | 1 |
| **6** | Asks patient what pronoun should be used when writing clinical note | 3 | 2 | 1 |
| **HISTORY** | | **Competently Performed (3)** | **Needs Improvement (2)** | **Did Not Attempt (1)** |
| **7** | Begins the HPI with open-ended questions | 3 | 2 | 1 |
| **8** | Negotiates agenda/focus of visit | 3 | 2 | 1 |
| **9** | Elicits patient's perspective and beliefs (fears, concerns, ideas) | 3 | 2 | 1 |
| **10** | Facilitates the storytelling by saying, "Tell me more" | 3 | 2 | 1 |
| **COMMUNICATIONS SKILLS THROUGHOUT THE ENCOUNTER** | | **Competently Performed (3)** | **Needs Improvement (2)** | **Did Not Attempt (1)** |
| **11** | Uses nonverbal skills to demonstrate that they are attentive and present (brings Kleenex) | 3 | 2 | 1 |
| **12** | Accommodates patient comfort | 3 | 2 | 1 |
| **13** | Maintains eye contact | 3 | 2 | 1 |
| **14** | Speaks clearly and audibly | 3 | 2 | 1 |
| **15** | Demonstrates active listening (includes non-verbals, headnodding, posture) | 3 | 2 | 1 |
| **16** | Demonstrates empathy when appropriate throughout the encounter | 3 | 2 | 1 |
| **17** | Avoids using medical jargon, or explains jargon throughout the encounter | 3 | 2 | 1 |
| **18** | Adjusts tone and pacing as appropriate throughout the encounter | 3 | 2 | 1 |
| **COMMUNICATION SKILLS: DELIVERING REALLY BAD NEWS (End of Life)** | | **Competently Performed (3)** | **Needs Improvement (2)** | **Did Not Attempt (1)** |
| **19** | Creates a mutual understanding of the patient’s prognosis | 3 | 2 | 1 |
| **20** | Delivers the bad news in very plain language, slowly and carefully | 3 | 2 | 1 |
| **21** | Allows for silence as appropriate | 3 | 2 | 1 |
| **22** | Responds appropriately to the patient's emotions | 3 | 2 | 1 |
| **23** | Gives information in small amounts | 3 | 2 | 1 |
| **24** | Effectively communicates both known details and uncertainty | 3 | 2 | 1 |
| **25** | Assures the patient that the medical team would continue to help the patient | 3 | 2 | 1 |
| **26** | Asks about the patient’s significant relationships/support system | 3 | 2 | 1 |
| **27** | Offers to partner with the patient in having difficult conversations with family | 3 | 2 | 1 |
| **28** | Considers cultural and other factors that influence the patient’s understanding of illness/diagnosis | 3 | 2 | 1 |
| **29** | Elicits and incorporates (if appropriate) the patient’s spiritual/religious beliefs into the discussion | 3 | 2 | 1 |
| **30** | Elicits the patient’s goals of care (i.e. "at this stage given your understanding of the situation what are your goals?") | 3 | 2 | 1 |
| **31** | Student demonstrated compassion - (i.e. apology followed by 'I wish things were different…') | 3 | 2 | 1 |
| **COMMUNICATIONS: SHARED DECISION MAKING and ASK TELL ASK** | | **Competently Performed (3)** | **Needs Improvement (2)** | **Did Not Attempt (1)** |
| **32** | Sets an agenda of what will be discussed - possible treatment plans | 3 | 2 | 1 |
| **33** | Assesses prior knowledge - 'do you know anyone else who has had this kind of serious diagnosis?' (ASK) | 3 | 2 | 1 |
| **34** | Gives information clearly, avoiding medical jargon (or explains jargon if used) (TELL) | 3 | 2 | 1 |
| **35** | PEARLS response^a^ | 3 | 2 | 1 |
| **36** | Asks what other information might be helpful (ASK) | 3 | 2 | 1 |
| **37** | Allows time for questions and concerns | 3 | 2 | 1 |
| **38** | Inquires if patient wants to include someone else next visit | 3 | 2 | 1 |
| **39** | Respect patient's period of indecision | 3 | 2 | 1 |
| **40** | Demonstrates respect for the patient’s autonomy | 3 | 2 | 1 |
| **41** | Ends the encounter with a clear follow up plan | 3 | 2 | 1 |
| **CLOSES INTERVIEW** | | **Competently Performed (3)** | **Needs Improvement (2)** | **Did Not Attempt (1)** |
| **42** | Summarizes visit's important points and uses TEACH BACK (i.e. when you are talking to your family tonight and they ask about the visit, what will be your response?) | 3 | 2 | 1 |
| **43** | Thanks the patient and uses one more PEARLS statement to close the visit, before standing up^a^ | 3 | 2 | 1 |
| **44** | Sanitizes hands at the end of the encounter on the way out | 3 | 2 | 1 |
| **IMPRESSIONS** | | | | |
| **45** | Was there any lapse in relationship-building skills? If yes, please elaborate in comment box | **YES** | **NO** |  |
| **46** | Was there anything of concern in the encounter? If yes, please elaborate in comment box | **YES** | **NO** |  |
| **Comments:** | | | | |

^a^PEARLS (Partnership, Empathy, Apology, Respect, Legitimization, and Support) is a communication skills tool developed by the Academy of Communication in Healthcare and used often as part of CWRU-SOM CW simulation didactics. For an example of its use see Wert K, Donaldson AM, Dinh TA, Montero DP, Parry R, Renew JR, Yip DS, Speicher L. Communication training helps to reduce burnout during COVID-19 pandemic. *Health Serv Res Manag Epidemiol*. 2023 Feb 2;10:23333928221148079. doi: 10.1177/23333928221148079.
