# Supplementary material for: Values and Uncertainty at End of Life: A Standardized Patient Case for Preclinical Medical Students
Source: MedEdPORTAL. 2025 Mar 4;21:11503. doi: 10.15766/mep_2374-8265.11503 (PMC11876469; doi:10.15766/mep_2374-8265.11503)
Supplement: Supplementary file 1 — SP Case.docxPeer Debrief Questions.docxDoor Note.docxStudent Self-Assessment.docxSP Assessment.docx [file mep_2374-8265.11503-s001.zip › _Educational Summary Report_11503.pdf]

MedEdPORTAL® | The AAMC Journal of Teaching and Learning Resources

Original Publication Open Access

Values and Uncertainty at End of Life: A Standardized Patient Case for Preclinical Medical Students

Ryan Jenkins, MD, Anastasia Rowland-Seymour, MD, Erin Gentry Lamb, PhD*

*Corresponding author: egl36@case.edu

Abstract

Introduction: Physician learners desire more and higher-quality education on end-of-life care. Challenges include the inherent difficulties of clinical uncertainty and how to provide meaningful experiences for early learners. This standardized patient (SP) encounter features a patient facing a newly terminal diagnosis. The patient's goals and values are not specific, and the encounter has an open-ended resolution. This low-stakes formative exercise was administered to preclinical medical students. **Methods:** Designed as part of a broader research project on using humanities to teach end-of-life care to medical students, our case was administered to 178 second-year students (98% of the class) as a required part of their simulation curriculum. Students ($n = 171$, 96%, consented for research participation) and SPs answered posttest Likert-scale questions assessing student comfort, confidence, and performance during the encounter. Students ($n = 175$, 96% of the class) later provided feedback as part of an overall curriculum evaluation. **Results:** Students found the case anxiety-provoking ($M = 4.8/7.0$) but felt comfortable while performing ($M = 4.7/7.0$) and expressed confidence in their ability to admit uncertainty ($M = 5.7/7.0$). SPs found students performed well at eliciting goals of care ($M = 5.8/7.0$) and creating plans ($M = 6.3/7.0$). On retrospective evaluation, students felt the case accessed the uncertainty learning objective (98% agreed or strongly agreed). **Discussion:** The case feasibly targeted the uncertainty- and prognosis-related learning objectives. Students found it challenging but performed well and rated their experience positively. We discuss overall strengths and areas for improvement as well as options for future implementation.

Keywords
Competency-Based Medical Education, End-of-Life/Palliative Care, Standardized Patient

Educational Objectives

By the end of this activity, second-year medical student learners will be able to:

1. Discuss goals and values with a patient facing a terminal diagnosis.
2. Determine a reasonable plan/next step for a patient in the setting of clinical uncertainty.
3. Communicate professionally and empathetically with a patient facing a new diagnosis of a terminal illness.

Citation:
Jenkins R, Rowland-Seymour A, Lamb EG. Values and uncertainty at end of life: a standardized patient case for preclinical medical students. *MedEdPORTAL*. 2025;21:11503.
https://doi.org/10.15766/mep_2374-8265.11503

Introduction

End-of-life care in medical education remains a significant area for growth. Medical students and new residents routinely report concerns with their provision of this care, including feeling unprepared and distressed,[1,2] finding cases challenging,[3,4] and desiring more exposure to improve their skills.[2,5] Clinical uncertainty and ambiguity, critical considerations when evaluating physiological and values-based aspects of death, have also been associated with negative affective states of fear, worry, and anxiety in health care contexts,[6] as well as with stress, burnout, and psychiatric diagnoses in medical trainees.[7] While most medical schools address death and dying in some way, little agreement exists on the ideal content and approach.[8] Even once established, these curricula can be unstandardized and underdeveloped, as well as lacking evidence of their efficacy.[2,9,10] Consistent with this broader lack of consensus on education, the appropriate stage of training to address end-of-life concerns also remains in contention.[1,4] The inherent challenges can be intensified for earlier learners, who, in addition to the

Copyright © 2025 Jenkins et al. This is an open-access publication distributed under the terms of the Creative Commons Attribution license.

above, often feel implicitly or explicitly unwelcome and excluded from palliative care cases.[11]

Simulation-based learning has the potential to address many of these concerns. It provides experiences on demand in a controlled environment when exposure to real cases may be limited by availability and of tempered benefit due to learner anxiety.[5,12] Given the emotional heft of end-of-life topics simulation also allows for a guaranteed debriefing opportunity that may not be feasible during busy clinical practice.[13] Simulation can be targeted at skill acquisition and development to provide foundational opportunities prior to using these skills in real encounters.[14] These strengths apply whether talking about high-fidelity technological simulations or more accessible standardized patient (SP) encounters. However, as with other areas of end-of-life care, the literature remains relatively sparse on simulation's use in teaching palliative care concepts.[15]

To address this need, we created an SP encounter that offers learners experience with these challenging aspects of discussing end-of-life care, particularly patient preference uncertainty and an emphasis on exploring patient values and hopes beyond the clinical facts of the diagnosis. We intended this encounter to be part of a foundational introductory experience for learners who have not yet acquired significant clinical time. Although existing simulations in the literature cover topics such as breaking bad news[16] and active dying,[17] fewer allow for an open-ended approach to exploring imminent mortality with a cognitively intact patient. Materials developed by Talwalkar and colleagues[18] include a case of a patient with newly terminal cancer; however, the patient in this case has clear ideas of what they want their end-of-life care to look like. Several authors have developed cases with similar clinical backgrounds but have learners approach them as a group instead of individually.[19,20] Furthermore, only one of these resources[20] targets preclinical learners.

As learners have readily identified, cases like ours carry a significant emotional burden distinct from other challenges faced in medical education, particularly given the degree of individuality and ambiguity our specific scenario asks participants to navigate. Accordingly, we assigned it to students in a low-stakes formative fashion as a communications workshop (CW) at the Case Western Reserve University School of Medicine (CWRU-SOM). CWs in the CWRU-SOM curriculum involve one-on-one student encounters with SPs as a bridge between student-to-student role-playing and directly-observed patient encounters in clinic, all part of an iterative process towards building communication skills comfortably and preparing students for clinical practice during clerkships.

Methods

Development

The case centered on a 45-year-old patient with a newly terminal diagnosis of glioblastoma multiforme. Students in the role of working with the patient's primary care doctor were tasked with discussing two options presented by the patient's oncologist: (1) chemotherapy that might provide a longer life expectancy but with burdensome side effects or (2) enrollment in hospice to prioritize comfort but with a shorter life expectancy. Neither the door note nor the SP instructions set an expectation that one of these options had to be selected by the end of the encounter; instead, SP instructions were explicit that a decision did not have to be made and did not specify which the patient preferred.

We initially conceptualized the encounter as part of a broader project evaluating the role of medical humanities in end-of-life education; other analyses related to this project will be published separately. Our team included a resident physician in categorical pediatrics who wrote the first draft of the case, a PhD in literature, and a practicing general internist (also CWRU-SOM director of preclerkship clinical skills development and assistant dean for longitudinal clinical education) who collaborated on revisions. We followed CWRU-SOM's in-house adaptation of the Association of Standardized Patient Educators Case Development Template,[21] which was used for all SP encounters at the institution. After agreeing on a draft, we pilot-tested the encounter with three SPs and six third- and fourth-year medical student volunteers, who were compensated $100 for their time. These testers completed the encounter and data-collection instrument drafts, then immediately provided in-person group feedback that we incorporated into the final materials (see Appendix A). All work was reviewed and exempted by the CWRU Institutional Review Board.

Implementation

SP recruitment and training followed CWRU-SOM standards. SPs received general onboarding training when recruited for the first time, and each also completed training specific to this case: two 2- to 4-hour sessions to review the materials and clarify questions, which both occurred in the 2 weeks prior to administration. Also, as was standard for CWRU-SOM CWs, fourth-year medical students on a near-peer teaching elective observed and provided individual feedback to the participants based on a standard checklist for CWs focusing on giving bad news (Appendix B); their interactions have not been included in

Copyright © 2025 Jenkins et al. This is an open-access publication distributed under the terms of the Creative Commons Attribution license.

our analysis. Observers worked with groups of four participants at a time; thus, participating students also observed three of their peers complete the encounter, sometimes before completing the encounter themselves.

Medical students completed our encounter as a required part of their simulation curriculum towards the end of their second-year coursework, which we targeted because their didactic knowledge foundation was almost complete but they had yet to participate in clinical experiences of significant depth. Their concurrent didactic curriculum included problem-based learning cases on neurologic chronic illness and brain death, as well as online modules on giving bad news[22] and shared decision-making.[23] Forty-one students completing the CW (23% of the class of 182) had earlier in the year completed an elective course on end-of-life care taught with humanities pedagogy as part of our overall research project, analyses of which will be published separately. CWs generally supplemented broader communications skills training in the CWRU-SOM curriculum; our focus here is solely on the SP encounter.

One hundred seventy-eight students (98%) of the second-year class of 182 participated in the encounter, and 171 (96%) consented to participate in our analysis. Students completed the encounter in a simulation center clinic room environment. Personnel included SPs, observers, and one administrator coordinating logistics for each session. Only this encounter was assigned for the day. Prior to the date of the workshop, students received informed consent materials for the use of student and SP responses in this research, which they reviewed with one of the authors and signed immediately before each CW session.

Upon arrival at the simulation center, students had the opportunity to ask questions of the administrator and then proceeded to their assigned room. They had 20 minutes to complete the encounter, including reviewing a door note (Appendix C). No physical exam or simulated notes or orders were required. Afterwards, students left the room and had 5 minutes to complete an electronic self-assessment (Appendix D) and a further 5 minutes of debrief with their observer and peers. SPs completed their own assessment (Appendix E) electronically inside the room during the latter 10 minutes.

Learner Assessment

With the expectation that learners' knowledge, attitudes, emotions, and skills would all directly correlate with their performance during the simulated encounter, we devised multipart assessments of the encounter filled out both by the students and the SPs. Students completed de novo reaction-level questions about their perceived end-of-life skills and how this SP encounter compared to other SP-based CWs on a 7-point Likert scale (1 = *strongly disagree,* 2 = *disagree,* 3 = *somewhat disagree,* 4 = *neither agree nor disagree,* 5 = *somewhat agree,* 6 = *agree,* 7 = *strongly agree*). Of note, students also completed two additional scales that we utilized specifically for a comparative analysis that will be published separately; therefore, we have excluded them from the materials provided here. SPs first completed items from CWRU-SOM's standard CW assessment form, followed by a set of de novo questions we wrote to measure students' end-of-life skills on the same 7-point Likert scale as above. Our de novo questions drew loosely on concepts from the Kalamazoo Essential Elements Communication Checklist–Adapted[24] and the Association of American Medical Colleges' Core Entrustable Professional Activities for Entering Residency.[25]

Additionally, CWRU-SOM regularly solicited student feedback on the overall curriculum. With respect to this specific CW, students answered the following questions at the end of their clinical block (approximately 2 months after the CW administration):

- End-of-Life Communications Workshop allowed me to demonstrate providing care in the face of uncertainty.
- Communication workshops prepared me to gather a history and demonstrate advanced patient-doctor communication skills.

These responses were scored on a 4-point Likert-type scale (1 = *strongly disagree,* 2 = *disagree,* 3 = *agree,* 4 = *strongly agree*). One hundred seventy-five students responded to the follow-up questionnaire (96% of the class). Throughout our analysis, we utilized descriptive statistics for all assessments.

Results

Results of student self-assessments are presented in Table 1. Overall, students reported feeling comfortable ($n = 105$, 61%, at least somewhat agreeing; $M = 4.7/7.0$) but also anxious ($n = 121$, 71%, at least somewhat agreeing; $M = 4.8/7.0$) while performing the encounter. However, many felt less comfortable ($n = 66$, 39%, at least somewhat disagreeing; $M = 4.0/7.0$) and more anxious ($n = 91$, 53%, at least somewhat agreeing; $M = 4.5/7.0$) as compared to other SP encounters at our institution. Despite this, they felt confident in their ability to admit uncertainty to a patient ($n = 149$, 87%, at least somewhat agreeing; $M = 5.7/7.0$) and that their encounter was valuable to the patient ($n = 157$, 92%, at least somewhat agreeing; $M = 5.8/7.0$). Results of SP assessments are presented in

Copyright © 2025 Jenkins et al. This is an open-access publication distributed under the terms of the Creative Commons Attribution license.

Table 1. Student Evaluation Responses (*N* = 171)

Questions	No. (%)							
	Strongly Agree	Agree	Somewhat Agree	Neutral	Somewhat Disagree	Disagree	Strongly Disagree	*M*[a]
I feel comfortable providing care to a patient when there are no curative treatments available.	15 (9)	46 (27)	56 (33)	13 (8)	31 (18)	9 (5)	1 (1)	4.8
I am confident I can discuss a patient's limited prognosis with them.	16 (9)	53 (31)	59 (35)	15 (9)	23 (13)	5 (3)	0 (0)	5.0
I am confident I can account for a patient's religious and spiritual beliefs and practices when discussing their terminal condition.	18 (11)	33 (19)	53 (31)	26 (15)	30 (18)	6 (4)	5 (3)	4.7
I am confident I can account for a patient's cultural beliefs and practices when discussing their terminal condition.	13 (8)	42 (25)	53 (31)	27 (16)	29 (17)	7 (4)	0 (0)	4.8
I am confident I can admit uncertainty to a patient.	42 (25)	74 (43)	33 (19)	12 (7)	8 (5)	2 (1)	0 (0)	5.7
The time I spent with the patient was valuable to them.	35 (20)	81 (47)	41 (24)	11 (6)	3 (2)	0 (0)	0 (0)	5.8
I felt comfortable during this OSCE.[b]	18 (11)	47 (27)	40 (23)	24 (14)	23 (13)	17 (10)	2 (1)	4.7
I felt more comfortable during this OSCE than other OSCEs I have taken.	13 (8)	29 (17)	25 (15)	38 (22)	26 (15)	26 (15)	14 (8)	4.0
I felt anxious during this OSCE.	17 (10)	38 (22)	66 (39)	17 (10)	18 (11)	14 (8)	1 (1)	4.8
I felt more anxious during this OSCE than other OSCEs I have taken.	21 (12)	34 (20)	36 (21)	32 (19)	24 (14)	20 (12)	4 (2)	4.5

[a]Scored on a 7-point scale (1 = *strongly disagree,* 2 = *disagree,* 3 = *somewhat disagree,* 4 = *neutral,* 5 = *somewhat agree,* 6 = *agree,* 7 = *strongly agree*).
[b]Although our administration of the standardized patient encounter is more properly referred to as a communications workshop than an OSCE given its lack of summative assessment, we used OSCE in our student-facing questions given the frequent use of the term as referring to any standardized patient encounter.

Tables 2 and 3. In general, SPs rated students as competently performing tasks including eliciting goals of care (*n* = 140, 82%, at least somewhat agreeing; *M* = 5.8/7.0) and creating plans for next steps (*n* = 165, 97%, at least somewhat agreeing; *M* = 6.3/7.0). Of the standard questions, student performance was notably lower on eliciting the patient's perspective and beliefs (*n* = 113, 66%, competently performed; *M* = 2.6/3.0) and facilitating storytelling with an open-ended question (*n* = 81, 48%, competently performed; *M* = 2.2/3.0). Of the end-of-life skills questions, student performance was notably lower on asking about spirituality (*n* = 82, 48%, at least somewhat agreeing; *M* = 4.5/7.0) and considering cultural factors that influenced the patient (*n* = 74, 43%, at least somewhat agreeing; *M* = 4.8/7.0).

On the clinical block summative questionnaire, when asked whether the CW "allowed me to demonstrate providing care in the face of uncertainty," 0% (*n* = 0) strongly disagreed, 2% (*n* = 3) disagreed, 63% (*n* = 110) agreed, and 35% (*n* = 62) strongly agreed. When asked whether the CW "prepared me to gather a history and demonstrate advanced patient-doctor communication skills," 0% (*n* = 0) strongly disagreed, 2% (*n* = 3) disagreed, 46% (*n* = 81) agreed, and 52% (*n* = 91) strongly agreed.

Note that given the slightly less than 100% participation in both the encounter and follow-up questionnaire, it is possible that a small subset of students responding to the questionnaire did not participate in the encounter. As a standardized instrument

Table 2. Standardized Patient Evaluation Responses to Standard Questions (*N* = 171)

Standard Question	No. (%)			
	Competently Performed	Needs Improvement	Did Not Attempt	*M*[a]
Establishes and maintains rapport with the patient.	145 (85)	26 (15)	0 (0)	2.8
Maintains eye contact.	156 (91)	15 (9)	0 (0)	2.9
Speaks clearly and audibly.	161 (94)	10 (6)	0 (0)	2.9
Demonstrates active listening (includes nonverbals, head nodding, posture).	147 (86)	24 (14)	0 (0)	2.9
Demonstrates empathy when appropriate throughout the encounter.	146 (85)	22 (13)	3 (2)	2.8
Avoids using medical jargon, or explains jargon throughout the encounter.	155 (91)	13 (8)	3 (2)	2.9
Adjusts tone and pacing as appropriate throughout the encounter.	128 (75)	42 (25)	1 (1)	2.7
Begins the history of present illness with open-ended questions.	152 (89)	19 (11)	0 (0)	2.9
Negotiates agenda/focus of visit.	147 (86)	24 (14)	0 (0)	2.9
Elicits patient's perspective and beliefs (fears, concerns, ideas).	113 (66)	52 (30)	6 (4)	2.6
Facilitates the storytelling by saying, "Tell me more."	81 (47)	54 (32)	36 (21)	2.2

[a]Scored on a 3-point scale (1 = *did not attempt,* 2 = *needs improvement,* 3 = *competently performed*).

Copyright © 2025 Jenkins et al. This is an open-access publication distributed under the terms of the Creative Commons Attribution license.

Table 3. Standardized Patient Evaluation Responses to Palliative Questions (N = 171)

	No. (%)							
**Palliative Question**	**Strongly Agree**	**Agree**	**Somewhat Agree**	**Neutral**	**Somewhat Disagree**	**Disagree**	**Strongly Disagree**	***M*[a]**
The student created a mutual understanding of the patient's prognosis.	86 (50)	62 (36)	16 (9)	0 (0)	5 (3)	1 (1)	1 (1)	6.3
The student delivered the bad news in very plain language, slowly and carefully.	84 (49)	45 (26)	30 (18)	2 (1)	9 (5)	1 (1)	0 (0)	6.1
The student allowed for silence as appropriate.	76 (44)	42 (25)	30 (18)	6 (4)	15 (9)	2 (1)	0 (0)	5.9
The student elicited the patient's goals of care.	74 (43)	42 (25)	24 (14)	11 (6)	16 (9)	3 (2)	1 (1)	5.8
The student ended the encounter with a clear plan for next steps (this does not necessarily mean that the patient and student decided which of the two treatment courses to pursue).	92 (54)	53 (31)	20 (12)	2 (1)	3 (2)	0 (0)	1 (1)	6.3
I am satisfied with the medical care the student provided.	90 (53)	47 (27)	22 (13)	2 (1)	6 (4)	2 (1)	2 (1)	6.2
The student responded appropriately to the patient's emotions.	95 (56)	42 (25)	27 (16)	1 (1)	3 (2)	2 (1)	1 (1)	6.3
The student assured the patient that the medical team would continue to help the patient.	131 (77)	32 (19)	6 (4)	0 (0)	1 (1)	0 (0)	1 (1)	6.7
The student offered to partner with the patient in having difficult conversations.	126 (74)	25 (15)	14 (8)	1 (1)	0 (0)	4 (2)	1 (1)	6.5
The student conducted the encounter with an honest, trustworthy demeanor.	122 (71)	44 (26)	3 (2)	1 (1)	1 (1)	0 (0)	0 (0)	6.7
The student elicited the patient's religious and/or spiritual beliefs and, if appropriate, incorporated these beliefs into the discussion.	41 (24)	31 (18)	10 (6)	36 (21)	6 (4)	32 (19)	15 (9)	4.5
The student asked about the patient's significant relationships/support system.	76 (44)	45 (26)	25 (15)	5 (3)	13 (8)	4 (2)	3 (2)	5.8
The student considered cultural and other factors that influenced the patient's understanding of illness/diagnosis.	31 (18)	38 (22)	5(3)	84 (49)	1 (1)	3 (2)	9 (5)	4.8
The student demonstrated respect for the patient's autonomy.	112 (65)	45 (26)	12 (7)	2 (1)	0 (0)	0 (0)	0 (0)	6.6
The student demonstrated compassion.	113 (66)	41 (24)	8 (5)	3 (2)	2 (1)	1 (1)	3 (2)	6.4
The student offered false hope that the condition is not imminently terminal.	10 (6)	0 (0)	5 (3)	4 (2)	11 (6)	50 (29)	91 (53)	2.0
The student effectively communicated both known details and uncertainty.	82 (48)	47 (27)	28 (16)	5 (3)	8 (5)	1 (1)	0 (0)	6.1

[a]Scored on a 7-point scale (1 = *strongly disagree,* 2 = *disagree,* 3 = *somewhat disagree,* 4 = *neutral,* 5 = *somewhat agree,* 6 = *agree,* 7 = *strongly agree*).

collecting data about many aspects of the curriculum, the questionnaire lacked the ability to capture this.

Discussion

We intended our case to fill a specific niche in the available SP encounter literature. Our script presents an unambiguously terminal diagnosis accessible without overwhelming detail to a preclinical learner, thereby allowing communication to focus more on questions about the patient's values and treatment preferences, which have been left deliberately nonspecific. Achievement of learning objectives suggests an acceptably high level of fidelity in a simulation,[26,27] which students participating in our case appreciated. By a large margin, the skill reflection questions the students most agreed with were that they could admit uncertainty to a patient and that the time they spent with the patient was valuable to the patient. When asked on the follow-up questionnaire whether the case allowed them to experience provision of care in the face of uncertainty, 98% of learners agreed or strongly agreed. We note that this level of agreement with the achievement of a learning objective is markedly higher than evaluations of other SP encounters at our institution, which, in the year prior to our encounter, ranged from 71% to 83%. Learners also showed high agreement that the encounter achieved the broader goals common to all CWs. Notably, these data also access assessments beyond the first Kirkpatrick level,[28] which is relatively uncommon in the palliative simulation literature.[14]

As discussed above, this type of encounter with patients facing the end of life can be distressing and emotionally challenging.[1-5] This is in part due to ambiguity itself, and given negative associations with poor ambiguity tolerance,[6,7] others have suggested that improved tolerance should be an education objective to ameliorate these concerns.[7] Our data support

Copyright © 2025 Jenkins et al. This is an open-access publication distributed under the terms of the Creative Commons Attribution license.

these findings given that many participants experienced anxiety, including many who rated their anxiety higher than other SP encounters. Likewise, many students expressed disagreement with feeling more comfortable during this encounter than other encounters, although we also note that a larger number still expressed at least some agreement with feeling comfortable during the encounter when considered in isolation.

Despite this anxiety and lower comfort, students displayed confidence in their ability to perform the tasks asked of them, with large numbers reporting agreement with each of the skills-based reflection questions. SPs also scored students highly when considering both standard CW performance items and novel items related to end-of-life care. In general, SPs rated large numbers of students as competent at most standard tasks, although we note that the most students needed improvement in eliciting the patient's perspectives and beliefs and facilitating the patient's own storytelling by requesting more information, items very central to the learning objectives. This speaks to a growth area we sought to access, as, in contrast to cases in the literature and other CWRU-SOM CWs, learning about the patient's goals via eliciting their thoughts and facilitating their storytelling was necessary in our case to help the patient start to choose between a number of potentially valid medical recommendations. SPs strongly agreed in most instances that students successfully addressed the end-of-life aspects of the case (or strongly disagreed when asked whether students offered false hope). The two questions for which results skewed less towards the desirable valence involved eliciting the patient's spirituality and considering their cultural beliefs, which, while generally important, were not explicitly telegraphed to students as objectives nor did we design the encounter such that they played critical roles in the patient's values system.

Collectively, these student self-assessments of comfort and confidence and SP assessments of student competence suggest that the encounter identified our learning objectives as targets for student growth while not going beyond the skill set of preclinical learners. CWRU-SOM SPs are trained to consider competence relative to the learner's stage in training. We also designed our assessments to capture some objectively observable behaviors that should not depend on judgment calls, thus allowing performance differences or deficits across the cohort to be identified where they exist. We can also surmise from the relatively high scores that the students' expected anxiety and discomfort did not derail any potential learning benefits for them, even though they did not have significant clinical experience.

Our choice to position this SP encounter as part of the broader CW curriculum afforded us methodological benefits and institutional buy-in; however, it also led to some limitations. Aspects such as the timing structure, having students in each debrief cohort observe their partners' attempts, and some elements of the assessment scales were all standardized elements of CW encounters. Students may have preferred or demonstrated different performance if given more time for what we intended to be a challenging interaction. The confounding factor of some students observing their peers prior to their own encounter may have affected subsequent performance, which we accepted as consistent with the formative low-stakes intentions of CWs overall. Likewise, learning opportunities for students might be increased through longer times for debrief. As noted by other authors,[14,15] expenses related to training and maintaining SP curricula can be significant, and indeed, our case's development and administration were supported by grant funding.

This interpretation of our data, while multimodal, lacks a control group or reference ranges beyond comparisons to other CWRU-SOM CWs discussed above, which cover very different topics; we also do not have baseline data capturing students' responses to the questions prior to the encounter, all of which limit the generalizability of our results. The strength of our SP-generated scores must always be considered in the context of bias. In addition to general impulses against providing negative feedback, we note that the uncertainty in our encounter was also novel to the SPs, who therefore may have tended to grade the encounter differently as a group. In acknowledgment of this, we have emphasized descriptive comparisons in our analysis as opposed to comparisons to other work based on statistical significance or effect size.

The initial running of the encounter demonstrated the feasibility of SP portrayal of and student response to simulation cases with deliberate clinical ambiguity. At the time of publication, our encounter has run two more times in the standard CWRU-SOM curriculum in a nonresearch context and continues to receive highly positive feedback. This case of an open-ended interaction with a patient facing a newly terminal diagnosis has potential applicability in many areas of a medical curriculum, including in contexts that could accommodate logistical adjustments aimed at improving student outcomes. Our analysis supports its use as a formative tool in a low-stakes environment. The encounter can also function as a summative assessment tool if positioned differently in the broader curriculum. The patient narrative itself could also be utilized outside the context of a

Copyright © 2025 Jenkins et al. This is an open-access publication distributed under the terms of the Creative Commons Attribution license.

time-limited one-on-one encounter or with learners beyond the preclinical level to achieve different learning goals. We encourage data collection related to any trialed adaptation of the case in different learner cohorts to contribute more evidence to the growing literature base supporting end-of-life medical education.

We present materials for a novel SP encounter dealing with uncertainty and patient values in the setting of a terminal diagnosis. When administered formatively to second-year medical students, our multimodal assessment suggests the encounter accessed and achieved our learning goals while being well received by participants.

Appendices

A. SP Case.docx

B. Peer Debrief Questions.docx

C. Door Note.docx

D. Student Self-Assessment.docx

E. SP Assessment.docx

All appendices are peer reviewed as integral parts of the Original Publication.

Ryan Jenkins, MD: Assistant Professor, Department of Pediatrics, University of Michigan Medical School; https://orcid.org/0009-0000-8499-6228

Anastasia Rowland-Seymour, MD: Associate Professor, Department of Internal Medicine, MetroHealth Medical Center; Associate Professor, Center for Medical Education, Case Western Reserve University School of Medicine

Erin Gentry Lamb, PhD: Associate Professor, Department of Bioethics, Case Western Reverse University School of Medicine

Acknowledgments
The authors wish to thank Lia Logio, Kelli Qua, Ever Mkonyi, and Vanessa Earp for their contributions to the project.

Disclosures
None to report.

Funding/Support
Funding and support for this research were provided by a grant to Dr. Erin Gentry Lamb, awarded in January 2021, from the Association of American Medical Colleges' Fundamental Role of Arts and Humanities in Medical Education initiative and the National Endowment for the Humanities: Democracy demands wisdom.

Ethical Approval
The Case Western Reserve University Institutional Review Board reviewed this project.

References

1. Sikstrom L, Saikaly R, Ferguson G, Mosher PJ, Bonato S, Soklaridis S. Being there: a scoping review of grief support training in medical education. *PLoS One*. 2019;14(11):e0224325. https://doi.org/10.1371/journal.pone.0224325
2. Head B, Schapmire T, Earnshaw L, et al. Improving medical graduates' training in palliative care: advancing education and practice. *Adv Med Educ Pract*. 2016;7:99-113. https://doi.org/10.2147/AMEP.S94550
3. Brighton LJ, Koffman J, Hawkins A, et al. A systematic review of end-of-life care communication skills training for generalist palliative care providers: research quality and reporting guidance. *J Pain Symptom Manage*. 2017;54(3):417-425. https://doi.org/10.1016/j.jpainsymman.2017.04.008
4. Smith-Han K, Martyn H, Barrett A, Nicholson H. "That's not what you expect to do as a doctor, you know, you don't expect your patients to die." Death as a learning experience for undergraduate medical students. *BMC Med Educ*. 2016;16:108. https://doi.org/10.1186/s12909-016-0631-3
5. Corvetto MA, Taekman JM. To die or not to die? A review of simulated death. *Simul Healthc*. 2013;8(1):8-12. https://doi.org/10.1097/SIH.0b013e3182689aff
6. Hillen MA, Gutheil CM, Strout TD, Smets EMA, Han PKJ. Tolerance of uncertainty: conceptual analysis, integrative model, and implications for healthcare. *Soc Sci Med*. 2017;180:62-75. https://doi.org/10.1016/j.socscimed.2017.03.024
7. Hancock J, Mattick K. Tolerance of ambiguity and psychological well-being in medical training: a systematic review. *Med Educ*. 2020;54(2):125-137. https://doi.org/10.1111/medu.14031
8. Schwartz AW, Abramson JS, Wojnowich I, Accordino R, Ronan EJ, Rifkin MR. Evaluating the impact of the humanities in medical education. *Mt Sinai J Med*. 2009;76(4):372-380. https://doi.org/10.1002/msj.20126
9. Fitzpatrick D, Heah R, Patten S, Ward H. Palliative care in undergraduate medical education—how far have we come? *Am J Hosp Palliat Care*. 2017;34(8):762-773. https://doi.org/10.1177/1049909116659737
10. Lippe M, Johnson B, Mohr SB, Kraemer KR. Palliative care educational interventions for prelicensure health-care students: an integrative review. *Am J Hosp Palliat Care*. 2018;35(9): 1235-1244. https://doi.org/10.1177/1049909118754494
11. Hawkins A, Tredgett K. Use of high-fidelity simulation to improve communication skills regarding death and dying: a qualitative study. *BMJ Support Palliat Care*. 2016;6(4):474-478. https://doi.org/10.1136/bmjspcare-2015-001081

Copyright © 2025 Jenkins et al. This is an open-access publication distributed under the terms of the Creative Commons Attribution license.

12. Smith MB, Macieira TGR, Bumbach MD, et al. The use of simulation to teach nursing students and clinicians palliative care and end-of-life communication: a systematic review. *Am J Hosp Palliat Care*. 2018;35(8):1140-1154. https://doi.org/10.1177/1049909118761386

13. Leighton K. Death of a simulator. *Clin Simul Nurs*. 2009;5(2): e59-e62. https://doi.org/10.1016/j.ecns.2009.01.001

14. Kozhevnikov D, Morrison LJ, Ellman MS. Simulation training in palliative care: state of the art and future directions. *Adv Med Educ Pract*. 2018;9:915-924. https://doi.org/10.2147/AMEP.S153630

15. Evans L, Taubert M. State of the science: the doll is dead: simulation in palliative care education. *BMJ Support Palliat Care*. 2019;9(2):117-119. https://doi.org/10.1136/bmjspcare-2018-001595

16. Cannone D, Atlas M, Fornari A, Barilla-LaBarca ML, Hoffman M. Delivering challenging news: an illness-trajectory communication curriculum for multispecialty oncology residents and fellows. *MedEdPORTAL*. 2019;15:10819. https://doi.org/10.15766/mep_2374-8265.10819

17. Hayley DC, Kalender-Rich JL, Mack J, Swagerty D. Development of a hybrid simulated patient experience to practice care of the dying older adult. *MedEdPORTAL*. 2018;14:10777. https://doi.org/10.15766/mep_2374-8265.10777

18. Talwalkar JS, Fortin AH, Morrison LJ, et al. An advanced communication skills workshop using standardized patients for senior medical students. *MedEdPORTAL*. 2021;17:11163. https://doi.org/10.15766/mep_2374-8265.11163

19. Jeffers JM, Bord S, Hooper JE, Fleishman C, Cayea D, Garibaldi B. End-of-life care: a multimodal and comprehensive curriculum for graduating medical students utilizing experiential learning opportunities. *MedEdPORTAL*. 2021;17:11149. https://doi.org/10.15766/mep_2374-8265.11149

20. Croft C, Pletcher J, Fulmer VL, Steele RCT, Day H, Spagnoletti CL. A same-sex couple copes with end-of-life issues: a case materials guide. *MedEdPORTAL*. 2013;9:9438. https://doi.org/10.15766/mep_2374-8265.9438

21. ASPE's case development template. Association of Standardized Patient Educators. Accessed February 12, 2025. https://www.aspeducators.org/aspe-case-development-template

22. Quill T, Dennis C, Caprio A, Gracey C. Sharing serious news. Drexel University College of Medicine Center for Professionalism and Communication in Health Care. Accessed February 12, 2025. https://webcampus.med.drexel.edu/PCHC/About/FreeModules/DeliveringSeriousNews/Default.html

23. Lown B. Share information. Drexel University College of Medicine Center for Professionalism and Communication in Health Care. Accessed January 7, 2022. https://webcampus.drexelmed.edu/doccom/db/read.aspx

24. Rider EA, Nawotniak RH. *A Practical Guide to Teaching and Assessing the ACGME Core Competencies*. 2nd ed. HCPro; 2010:1-137.

25. *Core Entrustable Professional Activities for Entering Residency: Curriculum Developers' Guide*. Association of American Medical Colleges; 2014. Accessed February 12, 2025. https://store.aamc.org/downloadable/download/sample/sample_id/63/%20

26. Hamstra SJ, Brydges R, Hatala R, Zendejas B, Cook DA. Reconsidering fidelity in simulation-based training. *Acad Med*. 2014;89(3):387-392. https://doi.org/10.1097/ACM.0000000000000130

27. Kim J, Park JH, Shin S. Effectiveness of simulation-based nursing education depending on fidelity: a meta-analysis. *BMC Med Educ*. 2016;16:152. https://doi.org/10.1186/s12909-016-0672-7

28. Kirkpatrick DL, Kirkpatrick JD. *Evaluating Training Programs: The Four Levels*. 3rd ed. Berrett-Koehler; 2006.

Received: August 26, 2024
Accepted: January 3, 2025
Published: March 4, 2025

Copyright © 2025 Jenkins et al. This is an open-access publication distributed under the terms of the Creative Commons Attribution license.
